# Buckwheat Starch Isolated From Varieties Grown in Washington State: A Qualitative Characterization

**DOI:** 10.1111/1750-3841.70219

**Published:** 2025-05-07

**Authors:** Shweta Suri, Aniket Kamboj, Xiaofeng Guo, Kevin M. Murphy, Girish M. Ganjyal

**Affiliations:** ^1^ School of Food Science Washington State University Pullman Washington USA; ^2^ Department of Chemistry Washington State University Pullman Washington USA; ^3^ Department of Crop and Soil Sciences Washington State University Pullman Washington USA

**Keywords:** buckwheat, gelatinization, morphology, native buckwheat starch, pasting, starch

## Abstract

Buckwheat, a gluten‐free pseudocereal with high starch content, has great potential for use in numerous food product applications. Variability in the technofunctional properties across varieties is not well understood. Starch was isolated from two varieties of common buckwheat, Koto (KB) and Tinker (TB), cultivated in Washington State. Analysis was conducted on the chemical, functional, thermal, pasting, and morphological attributes of the isolated buckwheat starch. The findings exhibited great purity of starch (85.21%–89.31%) with high amylose content (23.06%–25.04%), minor ash (0.17%–0.42%), and protein fractions (1.36%–1.55%) exhibiting statistically significant differences among the varieties. Buckwheat starch micrographs showed the presence of polygonal, spherical, and round granules with smooth surfaces. Buckwheat starch granules displayed a smaller size (1.84–14.60 µm) with an average size of 6.35–6.68 µm. The solubility as well as swelling power of starch showed an increasing trend with rising temperatures from 30 to 90°C for both varieties. Both buckwheat varieties showed similar peak positions on their thermographs, ranging from 70.31 to 71.11°C. The greater starch peak viscosity of Koto buckwheat starch (743.55 ± 0.91 BU) was associated with starch granule sizes. Pearson correlation analysis exhibited a strong positive correlation among starch content and peak viscosity, breakdown viscosity, setback viscosity, end‐of‐cooling period viscosity, and amylose content. Nonetheless, there was an inverse association between starch content as well as water solubility, swelling power, and water absorption capacity. The findings revealed favorable functional and pasting attributes of buckwheat starches, indicating potential usage across a range of food products, such as bakery products, ready‐to‐eat meals, and frozen foods.

## 1 Introduction

Buckwheat is a type of annual dicotyledonous pseudocereal from the *Polygonaceae* family, which consists of 22 species. The two primary species grown for human consumption are common buckwheat (*Fagopyrum esculentum* Moench) as well as Tartary buckwheat (*Fagopyrum tataricum* (L.). Gaertn.) due in part to their broader adaptability in various growing conditions (Jha et al. 2024). According to Zhu (2016a), buckwheat can be cultivated in unfertile marginal lands with extreme weather conditions plus poor soil quality. It is adapted to regions with mild and damp climates that receive regular rainfall during the growing season. It is grown as a cover crop and appreciated for its efficiency in cultivation and ability to suppress weeds in a crop rotation during the summer (Breslauer et al. 2023a). Different common names assigned to buckwheat have been utilized to track its movement across Asia and Europe and continue to be used to validate the source of buckwheat (Zhou et al. 2018). Its production decreased in the 20th century due to the competition with other staple cereals like wheat, but lately, its production has increased owing to its re‐identified functional capabilities (Ahmed et al. 2014). In 2020, the global buckwheat production reached 1.80 million tons, rising to 2.23 million in 2022 (FAOSTAT 2024). In the United States, buckwheat is predominantly grown in North Dakota, Washington, and Minnesota. Buckwheat farming is limited in western Washington State yet shows potential as a summer crop that provides a short‐season alternative to staple cereal grains (Breslauer et al. 2023b).

Buckwheat is abundant in essential nutrients including starch (65.6%–76.8%), protein (6.8%–17.9%), fiber (0.8%–10.6%), vitamins, minerals, and bioactive compounds. Due to the growing focus on natural and nutritious foods, buckwheat is commonly included in functional foods for its gluten‐free and hypoglycemic properties (Yang et al. 2019; Breslauer et al. 2023b). Specifically, buckwheat may play a vital role in the gluten‐free diet for individuals with celiac disease (Giménez‐Bastida et al. 2015). Research indicates that buckwheat‐based foods exhibit a markedly lower rate of in vitro starch hydrolysis in comparison to wheat‐based foods (Tamura et al. 2021). Additionally, bioactive phytochemicals such as flavonoids, including quercetin and rutin, found in buckwheat contribute to a reduced postprandial glycemic response in vivo (Luo et al. 2019). Suzuki et al. (2020), Hatcher et al. (2008), and Li and Zhang (2001) suggested that buckwheat could be utilized in the development of food items like noodles, bread, pasta, biscuits, baked goods, and fermented items like wine, beer, and vinegar, indicating promising opportunities in the food sector. Further investigation is needed on the components of buckwheat, particularly starch, which constitutes a significant part of the grain. This will allow for more effective utilization of buckwheat in the market.

Starch, a type of glucose polysaccharide, is the major portion of buckwheat, attributing to approximately 70%–91% (w/w) based on the variety (Qin et al. 2010). On average, buckwheat starch consists of approximately 25% amylose, which is similar to other cereal grains (20%–28%; Zhu 2016b) and 75% amylopectin (Qin et al. 2010). The structural characteristics of amylose as well as amylopectin are essential in defining the physicochemical and functional attributes of starch, that in turn impact its suitability for various industries (Gao et al. 2024). As per Huo et al. (2023), starch with low amylose content exhibits lower gelatinization temperatures, elevated pasting viscosity, increased stickiness, and diminished hardness. These properties collectively contribute to an improved eating quality of food products.

Like other types of cereal starches, native buckwheat starch can be employed extensively in food items as a binding agent, thickening agent, film former, and foaming agent (Li et al. 2014). When used as a pie filling, buckwheat starch showed a consistent texture similar to wheat, millet, and triticale starches (Zhu 2016a). Buckwheat starch possess a smaller granular size, ranging from 3 to 14 µm, in contrast to maize as well as potato starch. It exhibits lower gelatinization enthalpy and water solubility; however, it possesses higher gelatinization temperatures (Gao et al. 2016). Moreover, the compact nature of buckwheat starch granules provides it with numerous benefits for stabilizing emulsions, offering potential applications in food, cosmetics, pharmaceuticals, and various other sectors (Lin et al. 2023). Buckwheat starch particles are small in size, resembling lipid micelles, indicating they could be used as fat substitutes (Gregori and Kreft 2012).

In addition, the smaller size of buckwheat starch was considered to be appropriate for use as an additive in biocomposites (Zhu 2016a). Nanocrystals, derived from buckwheat starch through acid hydrolysis, were added to make starch films. The films produced showed reduced water penetration and a three‐phase moisture adsorption transition (Neethirajan et al. 2012). However, the market for buckwheat starch has remained underdeveloped owing to a lack of comprehensive studies on its technofunctional properties and potential applications. Exploring the technofunctional attributes of buckwheat starch and the relationship between structure and function is crucial for furthering its applications as a food ingredient.

This study investigates the variability in the technofunctional attributes of starches derived from two different buckwheat varieties, Koto (KB) and Tinker (TB), cultivated in Washington, United States. While buckwheat production in the Pacific Northwest is relatively modest, it holds potential as a weed‐suppressive crop within organic farming systems as well as serves as a short‐season substitute to traditional cereal grains. This research aimed to isolate starch from two varieties of common buckwheat namely; Koto (KB) and Tinker (TB) cultivated in Washington, United States. The chemical, functional, thermal, pasting, and morphological attributes of the starch isolated from the two varieties were determined, and a correlation between physicochemical, functional, and structural attributes was established.

## 2 Materials and Methods

### 2.1 Materials

Koto buckwheat (KB) and Tinker buckwheat (TB) were grown side by side in Vancouver, Washington in 2022. KB is a dark color, round, large seeded variety, with high test weight and yield as compared to traditional varieties namely; “Koban” and “Mancan” (Breslauer et al. 2023b). KB became available to growers in the United States in 2002 and was developed in a joint project between Cornell University, Ithaca, New York, and Kade Research. TB is a high‐yielding, early maturing, and short‐statured variety developed by researchers at Washington State University and released in 2024. Both these varieties are distinct in themselves.

The buckwheat whole seeds were ground using a cyclone sample mill (Model 3010‐030, UDY Corp., Fort Collins, CO, USA) with a 0.5 mm screen. The composition of KB and TB were analyzed and reported in results Section 3.1.

### 2.2 Isolation of Starch

Starch was obtained by following the standard wet milling procedure outlined by Zheng et al. (1997). Both KB and TB groats weighing 600 g were immersed in a solution containing 0.20% sodium bisulfite (Fisher Scientific ACS, No. S654500) at 4°C for 16 h. The soaked mass was filtered through a 1 mm sieve to collect the soaking water. Subsequently, the soaked buckwheat was subjected to high‐speed blending in a kitchen blender with a little amount of deionized water for 2 min. The resulting slurry was hand‐sieved using a set of US Standard test sieves with sizes of 600 µm, 300 µm, 125 µm, 75 µm, and 63 µm (Sieve No. 30, 50, 120, 200, and 230, respectively). The first sieving operation yielded buckwheat husk, while the second and third operations produced germ and fiber fractions, and the fourth operation yielded protein fraction. During the sieving process, 4% w/v sodium hydroxide was slowly introduced into the slurry passing through the 75 and 63 µm sieves. The starch filtrate was then rested to settle at 4°C for 16 h, after which the supernatant was carefully collected, and the starch sediment was mixed with deionized water following centrifugation for five times at 4000 rpm for 15 min. Following centrifugation, the pure starch as well as starch tailings were collected and subsequently dried overnight in a hot air dryer (Model# 41640729; VWR International Radnor, PA, USA) set at 45°C. Similarly, the soaking water, supernatant from washing, and starch water obtained after centrifugation were also dried up in a hot air dryer at 45°C. The dried starch obtained from Koto (KS) and Tinker (TS) was then milled to powdered form in a cyclone sample mill using a 0.5 mm screen and stored in an airtight zip‐lock package under refrigeration conditions until further use.

The yield of each fraction (starch, starch with tailings, husk, germ and fiber, protein, total solids in stepping water, washings, and supernatant secured after centrifugation) was estimated by dividing the total dried fraction with the initial amount of buckwheat groats (Wronkowska and Haros 2014).

### 2.3 Starch Characterization

#### 2.3.1 Chemical Composition

Buckwheat starch samples (KS and TS) were examined for their proximate composition. The moisture was analyzed using the hot air dryer (Model# 41640729; VWR International Radnor, PA, USA) following the American Association of Cereal Chemists International (AACCI 2005) method 44‐15.02. The ash content was measured using a muffle furnace (Model# F6010; Thermo Fisher Scientific, Asheville, NC, USA) set at 600°C for 12 h as per Association of Official Analytical Chemists (AOAC 2006) standards, while the protein content of starch samples was analyzed using the rapid N exceed‐Nitrogen and Protein Analyzer (Elementar Americas Inc., NY, USA) as per the AACC 46‐30.01 method. The fat content of starch samples was analyzed by extraction process using Soxhlet apparatus as per AACC (2005) method 30‐25.01 (Soxhlet Apparatus, Precision Scientific Inc., Illinois, USA). Total starch as well as amylose content were examined through the Total Starch Assay Kit and Amylose/Amylopectin Assay Kit (K‐TSTA‐50A 03/20, Megazyme Intl. Ireland, Bray, Co. Wicklow, Ireland) (Megazyme, 2024). The amylose kit utilized a modified method derived from the research of Yun and Matheson (1990). This approach leverages the formation of amylopectin complexes with the lectin concanavalin A (Con A) to provide an optional and effective means of measuring amylose in starch.

#### 2.3.2 Water Solubility and Swelling Power

The water solubility as well as swelling power of the KS and TS samples were determined following the methodology outlined by Gao et al. (2020). Approximately 150 mg of buckwheat starch samples were mixed with 5 mL of distilled water and subjected to heating in a shaking water bath at varying temperatures (30°C, 50°C, 70°C, and 90°C) for 30 min. Subsequently, the starch samples were allowed to cool down at room temperature and later centrifuged at 3000 × *g* for 20 min using a centrifuge (Model# 5804, Eppendorf, Hauppage, NY, USA). The resulting supernatant was carefully transferred to aluminum dishes and then dried at 105°C. The weights of the precipitated starch as well as the supernatant were then recorded. The solubility as well as swelling power of the starch samples were calculated using the subsequent Equations (1) and (2):

(1) $$\mathrm{Solubility}\;\left(\%\right)=\left[\frac{W_{2}}{W_{1}}\right]\times 100.$$

(2) $$\mathrm{Swelling}\;\mathrm{power}\;\left(\frac{g}{g}\right)=\left[\frac{W_{3}}{W_{1}-W_{2}}\right]\times 100,$$

where *W*_1_ stands for the weight of the dry buckwheat starch sample; *W*_2_ stands for the weight of the dried supernatant; and *W*_3_ stands for the weight of the precipitated buckwheat starch.

#### 2.3.3 Water and Oil Absorption Capacity

The water and oil absorption capacities of KS and TS were studied as per the methodology outlined by Gao et al. (2020). Specifically, 1 g of buckwheat starch sample was accurately weighed and placed in a 15 mL falcon tube. Subsequently, it was blended with 10 mL of oil or water and vortexed for 10 min. Following this, the mixture was then centrifuged at 4000 rpm for 20 min. The supernatant obtained after centrifugation was discarded, and the falcon tubes were inverted over a paper tissue until no further oil flowed out. The tube as well as its contents were then weighed. The water, as well as oil absorption capacity of the starch samples, were calculated as per the prescribed Equation (3).

(3) $$\mathrm{Water}\;\mathrm{or}\;\mathrm{Oil}\;\mathrm{Absorption}\;\mathrm{Capacity}\;\left(\%\right)=\left[\frac{W_{2}-W_{1}}{W_{1}}\right]\times 100.$$

where *W*_1_ stands for the weight of the dry starch sample and *W*_2_ stands for the weight of the mixture after absorption.

#### 2.3.4 Microscopic Analysis

Microscopic examination was done following the method as outlined by Li et al. (2017). Buckwheat starch samples were carefully spread onto carbon adhesive stubs. To mitigate charging effects during scanning electron microscopy (SEM), a sputter coater (Cressington Scientific Instruments, Watford, UK) was employed to put a thin layer of gold. This process was conducted at a current of 20 mA for a duration of 60 s. The surface morphology was determined by a Scanning Electron Microscope (Quanta 200F, FEI Company, Hillsboro, OR, USA). The scanning electron microscope was utilized in high‐vacuum mode, with the accelerating voltage carefully adjusted to 20.0 kV and a working distance of approximately 13.2 mm while maintaining a chamber pressure of 130 Pa during imaging. Detailed images were captured using an Everhart–Thornley detector (ETD) for secondary electron imaging at magnifications of approximately 2500 × (10 µm scale) and 500 × (50 µm scale).

#### 2.3.5 X‐Ray Diffraction Analysis

X‐ray diffraction (XRD) measurements of starch samples were conducted using the Rigaku Miniflex 600 X‐diffractometer (Rigaku Corporation, Tokyo, Japan) following the method outlined by Gao et al. (2016). The X‐ray patterns of the buckwheat starches were recorded at diffraction angles ranging from 5° to 50°. The scanning conditions used were as follows: the X‐ray radiation source was Cu Kα (Kb was removed by a Ni filter) with a current of 30 mA and a voltage of 40 kV. The scanning speed was established at 5° per min, with a step time of 2 s and a step size of 0.02.

#### 2.3.6 Thermal Properties

The thermal attributes (gelatinization and retrogradation) of buckwheat starches (KS and TS) were determined by a differential scanning calorimetry (DSC, TA Instruments, New Castle, DE, USA). A total of 2 mg (db) of starch sample was weighed in stainless‐steel pans (Perkin Elmer in Norwalk, CT, USA), blended with 6 µL of distilled water, sealed, and equilibrated overnight. The thermal analysis was conducted at temperatures ranging from 30°C to 150°C at a scanning rate of 10°C/min. Following the scanning process, starch samples were kept at 4°C for 1 week, then brought to room temperature for 1 h and rescanned under similar temperature settings to analyze the retrogradation properties (Pietrysiak et al. 2024; Rodriguez‐Sandoval et al. 2017). The onset temperature (*T*_o_), peak temperature (*T*_p_), conclusion temperatures (*T*_c_), and enthalpy change (Δ*H*_g_ and Δ*H*_r_) of gelatinization and retrogradation were computed using the TRIOS software from TA Instruments.

#### 2.3.7 Pasting Properties

The pasting attributes of buckwheat starches were analyzed using ViscoQuick (Model# 803600, Brabender GmbH & Co., South Hackensack, NJ, USA) as per the protocol outlined by Bernin et al. (2024). The starch samples (9.5 g, 8% wet basis) were mixed with 105 mL of distilled water and then stirred to form the slurry. The samples were heated in the stainless‐steel pan from 30°C to 93°C at a rate of 7.5°C/min, held at 93°C for 5 min, cooled from 93°C to 50°C at the rate of 7.5°C/min and held at 50°C for 1 min. The following pasting parameters were obtained from the viscosity curves namely; Peak viscosity, breakdown viscosity, setback viscosity, and final viscosity in Brabender Units (BU). The pasting temperatures were reported in degrees Celsius.

### 2.4 Statistical Analysis

The results are represented as means and standard deviations (mean ± SD) of experiments performed in triplicates. The three replicates were analyzed by one‐way analysis of variance (ANOVA) and a Tukey's test for checking the significant differences set at *p* < 0.05. SPSS (IBM SPSS statistics version 22.0, North Castle Drive, Armonk, NY, USA), as well as OriginPro 2024b (Origin version 10.1.5.132, OriginLab Corporation, Northampton, MA, USA), were used for analyzing the statistical data.

## 3 Results and Discussion

### 3.1 Composition of Buckwheat

The composition of the milled whole KB and TB flours were determined to be 76.43 ± 0.85% and 69.91 ± 0.58% of starch; 2.21 ± 0.04% and 2.33 ± 0.06% of ash; 10.14 ± 0.04% and 10.31 ± 0.02% of protein; and 2.60 ± 0.44% and 2.51 ± 0.39% of fat, respectively, analyzed as per the methods explained in Section 2.3.1. The values for starch, protein, and fat content of buckwheat flours obtained in the present study was consistent with the results reported by Zheng et al. (2023), who reported 76.91% starch content, 8.9% protein content, and 1.84% fat content in buckwheat flour. However, in contrast to our results Sofi et al. (2022) reported protein content of 12.30%, fat content of 3.80%, and ash content of 2.0%.

### 3.2 Isolation of Starch and Yield

The yield of starch fractions obtained after wet milling of different varieties of buckwheat is shown in Table 1. For KB, wet milling resulted in a total starch yield of 42.55% and yields of husk, protein, and germ/fiber fractions at 26.17%, 4.40%, and 1.79%, respectively. The total solids leached out in the steeping water, washing, and centrifuge water was 18.64%. On the other hand, TB yielded 44.50% total starch, with husk, protein, and germ/fiber fractions at 19.10%, 3.96%, and 1.89%, respectively. The total solids content in steeping water, washing, and centrifuge water was 21.35%. Starch was the significant fraction obtained from both buckwheat varieties, followed by husk, protein, and germ/fiber fractions. The difference in starch yield and pure starch content following wet milling fractions of KB and TB can be attributed to factors including sieving operation (Pietrzak and Kawa‐Rygielska 2021), and chemicals used during steeping, such as sulfite salts (Yang et al. 2005), starch content of the varieties (Ballester‐Sánchez et al. 2019), and drying temperatures (Malumba et al. 2009). Sieving operation is considered the main reason in this research for difference in the starch yield after wet milling process.

**TABLE 1:** Yield of different fractions recovered from wet milling of buckwheat varieties.

	Yield (g/100 g) of kernels in dry matter	
Wet milling fractions	Koto buckwheat (KB)	Tinker buckwheat (TB)
Total starch	42.55 ± 0.97^a^	44.50 ± 0.45^b^
Pure starch	38.30 ± 0.78^a^	34.11 ± 0.47^b^
Starch with trailings	4.25 ± 0.19^a^	10.39 ± 0.66^b^
Husk (Hull)	26.17 ± 0.26^a^	19.10 ± 1.41^b^
Germ and fiber	1.79 ± 0.06^a^	1.89 ± 0.24^a^
Protein	4.40 ± 0.33^a^	3.96 ± 0.39^a^
Total solids in steeping water, washing and centrifuge water	18.64 ± 0.28^a^	21.35 ± 1.27^a^
Total recovery	93.55 ± 3.05^a^	90.80 ± 1.45^a^

Data presented as mean ± standard deviation. Mean values within columns with different uppercase superscript letters are significantly different (*p* < 0.05).

Furthermore, Table 1 illustrates that both varieties exhibited significant levels of total solids in the steeping water, washing, and centrifuge water. This is probably attributable to the high soluble dietary fiber found in the buckwheat bran (approximately 6.4%; Ma et al. 2023), which exceeds that of wheat bran (2.5%–2.8%; Rainakari et al. 2016).

### 3.3 Chemical Composition of Starch

The chemical components of the isolated starches are documented in Table 2. The analysis reveals a significant variance in the chemical composition (ash, protein, starch, and amylose content) of the buckwheat starches. The ash content in KS and TS was 0.42% and 0.17%, respectively, within the reported range in the literature (0.12%–0.51%; Gao et al. 2023; Goel et al. 2020). The fat content in KS and TS was 0.71% and 0.98%, respectively. The protein content in KS and TS was 1.36% and 1.55%, respectively, consistent with the results of Zheng et al. (2023), where common buckwheat native starch exhibited 1.47% protein content. The compositional analysis revealed a starch content of 89.31% in KS and 85.21% in TS, which is in line with the starch content of 89.11% mentioned by Goel et al. (2020). These findings indicate that the procedure used for buckwheat starch isolation effectively separated starch and nonstarch components (protein, fat, and fiber) with high purity.

**TABLE 2:** Chemical composition of buckwheat starch (KS and TS).

Samples	Moisture (%)	Ash (%)	Fat (%)	Protein (%)	Starch purity (%)	Amylose (%)
Koto buckwheat starch (KS)	8.80 ± 0.10^a^	0.42 ± 0.06^a^	0.71 ± 0.21^a^	1.36 ± 0.00^a^	89.31 ± 0.05^a^	25.04 ± 0.74^a^
Tinker buckwheat starch (TS)	8.72 ± 0.13^a^	0.17 ± 0.04^b^	0.98 ± 0.26^a^	1.55 ± 0.02^b^	85.21 ± 0.30^b^	23.06 ± 0.37^b^

Data presented as mean ± standard deviation. Mean values within columns with different uppercase superscript letters are significantly different (*p* < 0.05).

The amylose content of KS and TS starches was 25.04% and 23.06%, respectively. These values fall within the reported range for common buckwheat native starch (21.4%–25.6%) analyzed using iodine‐binding colorimetric method (Lu and Baik 2015). However, they were more than the values stated by Christa et al. (2009; 17.12 ± 1.43%) using iodine‐binding colorimetric method and lower than those reported for native buckwheat starch (28.05 ± 0.23%) by prior defatting of starch before following iodine‐binding colorimetric method (Goel et al. 2020). The amylose content of buckwheat starches differs considerably among different studies, which could be ascribed to genetic factors as well as agronomic conditions, cousage of nitrogen along with sulfur fertilizers during cultivation (Gao et al. 2016, 2024), and the method used for estimating the amylose content (Yoshimoto et al. 2004). The amylose fractions found in buckwheat starch are commonly measured using methods such as the concanavalin A‐precipitation‐based method (used in this study), iodine‐binding colorimetric method, and iodine‐binding amperometric titration‐based method. In comparing the iodine‐binding colorimetric method and iodine‐binding amperometric titration‐based method, it is noteworthy that the concanavalin A‐precipitation method offers greater accuracy in determining amylose content by effectively mitigating the effects of amylopectin (Zhu 2016a).

### 3.4 Functional Properties

**FIGURE 1:**
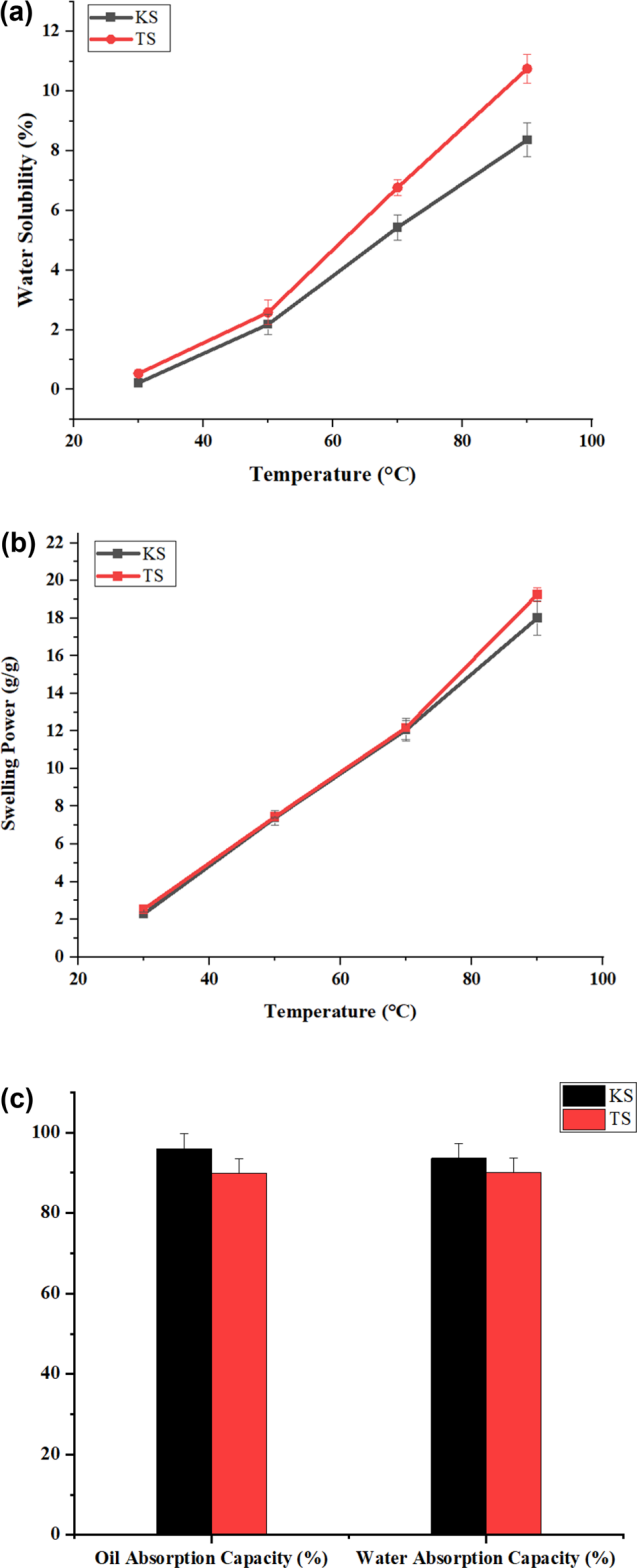
Functional properties of buckwheat starch (KS and TS). (a) Water solubility; (b) Swelling power; (c) Oil and water absorption capacities.

The functional attributes of buckwheat starches, including, solubility, swelling power, water, and oil absorption capacities, are presented in Figure 1a–c. Water solubility as well as swelling power are often utilized to evaluate the association among starch chains in both amorphous and crystalline regions. The water solubility of the KS and TS starches showed an increasing trend with increasing temperatures from 30 to 90°C. This could be because when starch molecules are subjected to high temperatures in excess water, the granules usually swell, disrupting the crystalline organization. Furthermore, water molecules create bonds with the exposed hydroxyl groups of amylose and amylopectin through hydrogen bonding, ensuing enhanced water solubility (Singh et al. 2003). The water solubility of KS was 0.22% at 30°C, 2.18% at 50°C, 5.42% at 70°C, and 8.36% at 90°C while the solubility of TS was significantly higher than KS with 0.53% at 30°C, 2.58% at 50°C, 6.76% at 70°C, and 10.75% at 90°C, respectively (Figure 1a). The swelling power of the KS and TS also followed the same trend as solubility. With increasing temperature from 30 to 90°C, there was a sharp increase in the swelling power of KS from 2.29 g/g to 18.00 g/g, respectively. Similarly, TS showed an increase from 2.54 g/g to 19.26 g/g at a temperature range of 30–90°C, respectively (Figure 1b). It is assumed that the rise in the temperature will decrease the intragranular adhesive forces of starch molecules, thereby allowing for more unrestricted swelling (Li et al. 2014).

The oil absorption capacity of KS and TS starches was 95.91 ± 0.97% and 89.96 ± 0.93%, respectively (Figure 1c), which was similar to the starches of two common buckwheat types namely; Xinong9976 and Pingqiao2 as reported by Gao et al. (2024). The water absorption capacity of KS and TS was 93.60 ± 0.17% and 90.04 ± 0.85%, correspondingly, which was in the accordance to water absorption capacity of native buckwheat starch (85.68 ± 0.74% to 98.83 ± 0.79%) as reported by Gao et al. (2020), however lower than the water absorption capacity of 138.24% reported by Goel et al. (2020).

### 3.5 Microscopic Analysis

The SEM images of the buckwheat starch (KS and TS) are presented in Figure 2. The micrographs showed the presence of polygonal, spherical, and round granules with smooth surfaces. Most of the granules exhibited irregular shapes with edges, which was coherent with findings reported in former research done on buckwheat starch (Sindhu and Khatkar 2023; Hu et al. 2022; Gao et al. 2023). Under high magnification, certain hollow spaces, small dents, and convexities were observed on the surface of the buckwheat starch granules, reminiscent of the patterns found in native protein bodies (Dura et al. 2014). The analysis of the microscopic image revealed that the buckwheat starch granules' size followed a normal distribution (Figure 2), and notable differences in granule size were observed between the two varieties. KS exhibited a slightly bigger size relative to TS. The granule size of buckwheat starches namely; KS and TS ranged from 1.84 to 14.60 µm, averaging 6.35–6.68 µm. The results are in line with those stated by Hu et al. (2022), who stated the diameter of native buckwheat starch ranges from 3 to 15 µm. Further, Wronkowska and Haros (2014) reported the granule size of starch ranging from 2 to 19 µm.

**FIGURE 2:**
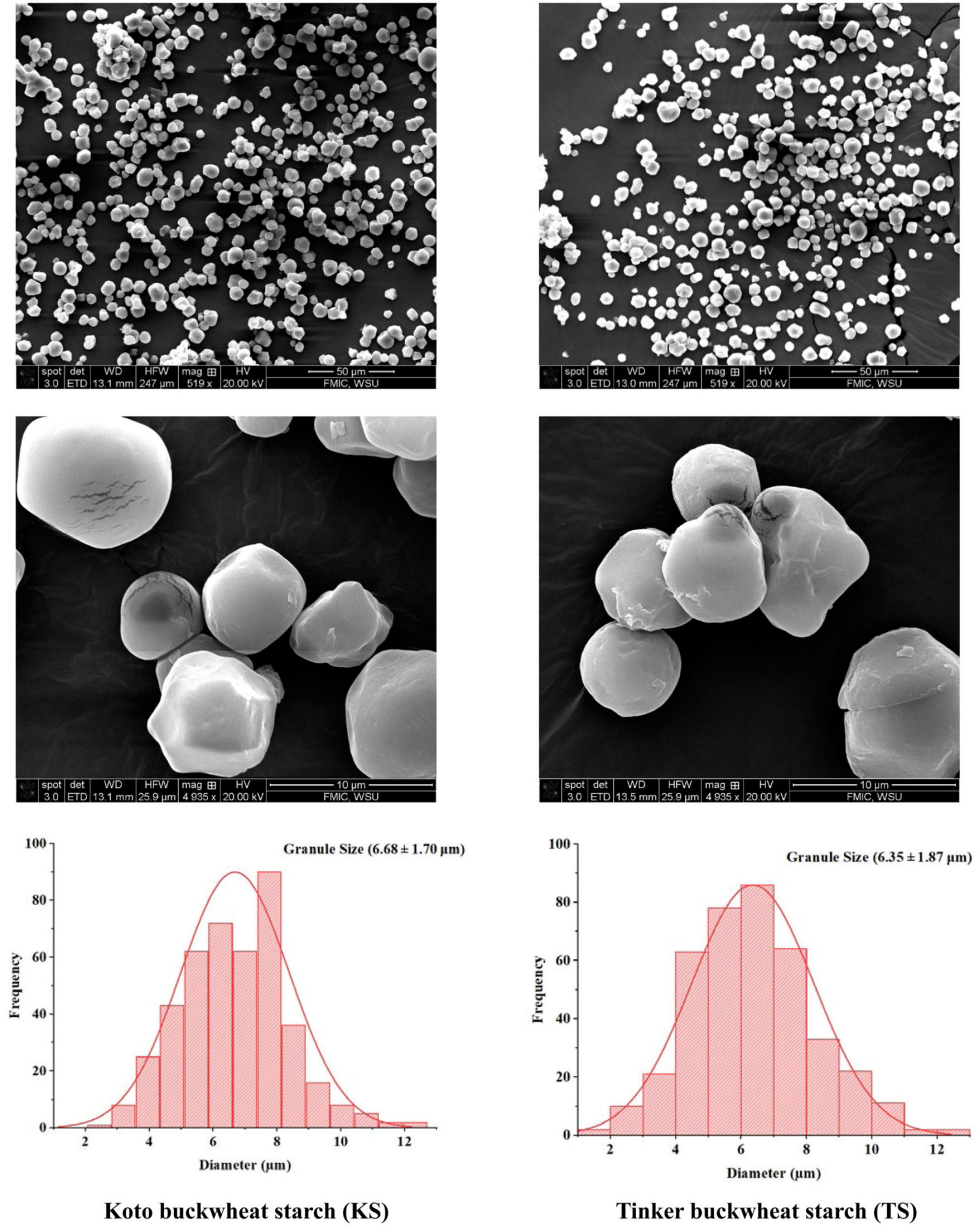
Scanning electron microscopy images and relative image analysis of buckwheat starch (KS and TS).

### 3.6 X‐Ray Diffraction Analysis

**FIGURE 3:**
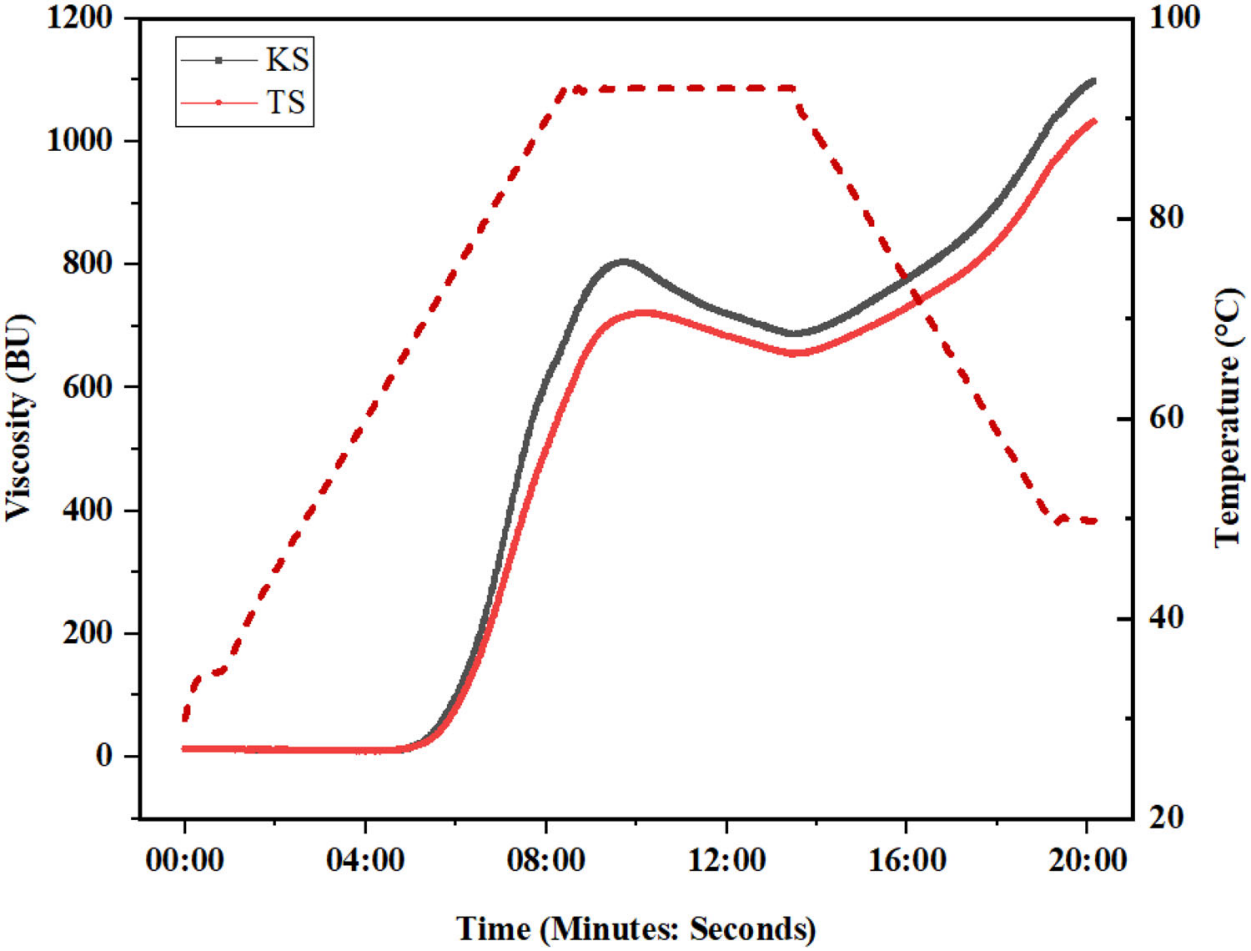
Viscosity profile of buckwheat starch (KS and TS).

XRD analysis was conducted to examine the crystalline structures of buckwheat starches (KS and TS, shown in Figure 3). Both KS and TS displayed typical A‐type crystalline peaks, with peaks of KS at 14.42°, 16.44°, 17.33°, and 22.34°, and those of TS at 14.30°, 16.40°, 17.28°, and 22.22°, in line with previously stated by Gao et al. (2016) and similar to patterns observed in cereal starches for instance wheat and rice (Zhang et al. 2013; Kim et al. 2013). The XRD patterns confirmed the regular and repetitive alignment of the ordered double helix structure, indicating the three‐dimensional organization of starch (Huang et al. 2022).

### 3.7 Thermal Properties

Studying the thermal attributes of starch is essential for understanding the standard cooking or processing conditions in developing products from starch or starch‐rich ingredients. The DSC data are presented in Table 3. According to the DSC analysis, the gelatinization starch temperature range for KS was 63.63–86.32°C, while for TS, it was 64.38–82.30°C. KS and TS starch samples exhibited smooth thermographs with comparable peak values at 71.11°C and 70.31°C, respectively. A statistically significant difference (*p* < 0.05) was noticed in the gelatinization temperature and enthalpy of KS and TS. The gelatinization process of starch is affected by various interactive factors, such as morphology of starch granule, amylose content in starch, molecular organization of amylopectin and amylose, as well as the content of minor components, all of which distinguish buckwheat starch from different types of cereal starches (Srichuwong and Jane 2007).

**TABLE 3:** Gelatinization and retrogradation properties of buckwheat starch (KS and TS).

Properties	Buckwheat starch	
	KS	TS
**Gelatinization**		
*T*_o_ (°C)	63.63 ± 0.79^a^	64.38 ± 0.56^a^
*T*_p_ (°C)	71.11 ± 0.22^a^	70.31 ± 1.32^a^
*T*_c_ (°C)	86.32 ± 0.97^a^	82.30 ± 0.90^b^
Δ*H*_g_ (J/g)	10.36 ± 0.12^a^	8.94 ± 0.14^b^
**Retrogradation**		
*T*_o_ (°C)	41.44 ± 1.35^a^	39.42 ± 0.55^b^
*T*_p_ (°C)	50.90 ± 0.65^a^	49.74 ± 0.64^b^
*T*_c_ (°C)	67.91 ± 0.93^a^	71.52 ± 0.89^b^
Δ*H*_r_ (J/g)	3.27 ± 0.93^a^	3.29 ± 0.58^a^

Data presented as mean ± standard deviation. Mean values within the same row with different uppercase superscript letters are significantly different (*p* < 0.05).

Abbreviations: *T*_c,_ concluding temperature; *T*_o,_ *T*_p,_ *T*_c_, transition temperatures (*T*_o_, onset temperature; *T*_p_, peak temperature); Δ*H*_g_ (J/g), enthalpy change.

Gelatinization enthalpy (Δ*H*_g_) indicates the energy needed to melt starch granules. A larger value signifies a higher energy demand for melting starch granules (Gao et al. 2016). Furthermore, the gelatinization enthalpies (Δ*H*_g_) are contingent upon the size of starch granules, hydration rates, and the interaction among different components. Notably, KS exhibited significantly higher Δ*H*_g_, attributed to its high starch content (89.31%) compared to TS (85.21%), indicating a greater energy requirement to unravel the intricate starch structure present in KS. Typically, starches exhibiting a higher degree of crystallinity are associated with greater Δ*H*_g_ values. The gelatinization of starch is an endothermic process that causes the disruption of crystallinity within starch granules when subjected to specific conditions of heat and moisture (Kadam et al. 2015). A comparative analysis of various cereal and tuber starches reveals distinct Δ*H*_g_ values, particularly in relation to buckwheat starch. For instance, wheat starch has a Δ*H*_g_ of 12.6 ± 0.09 J/g, corn starch is measured at 10.3 ± 0.01 J/g, waxy corn starch at 13.4 ± 0.18 J/g, potato starch at 14.9 ± 0.26 J/g, and sweet potato starch at 10.2 ± 0.81 J/g (Bajaj et al. 2022).

Furthermore, the existing literature expounds on the varietal distinctions that result in divergent peak gelatinization temperatures. Specifically, the peak gelatinization temperature of isolated buckwheat starch is documented to range from 62.29 to 63.75°C in Xinong9976 and Pingqiao2 variety (Gao et al. 2020), 66.20–66.80°C in Tartary buckwheat (Xinong9920 and Xinong9940; Gao et al. 2016); 76.50 ± 0.11°C in common buckwheat‐Yuqiao 4# (Liu et al. 2016); 66.09 ± 0.64°C in local buckwheat variety from China (Li et al. 2014), and 74.20°C in common buckwheat from Poland (Wronkowska and Haros 2014).

The retrogradation pattern of buckwheat starches outlined in Table 3 indicates the endothermic shift observed when the stored starch sample is reheated. The development of ordered starch during cold storage results from the re‐crystallization of amylopectin fractions of starch (Rodriguez‐Sandoval et al. 2017). In contrast to gelatinization, retrogradation endotherm displayed a narrower temperature range: 41.44–67.91°C for KS and 39.42–71.52°C for TS. Moreover, the retrogradation (Δ*H*_r_) enthalpies for KS and WS were notably lower than that of gelatinized starch. It shows the amount of energy needed to melt recrystallized amylopectin and measures the transition temperatures of endothermic events (Abd Karim et al. 2000).

### 3.8 Pasting Properties

Understanding the pasting attributes is essential as it influences the usage of starch in various food products. The pasting profile of buckwheat starches tested is presented in Figure 4 and Table 4. A notable statistical difference was observed in the peak viscosities, where KS expressed higher peak viscosity (743.55 ± 0.91 BU) compared to TS (702.20 ± 0.28 BU). Literature states that starches with bigger granule sizes occupy more space, which raises peak viscosity (Dong et al. 2021; Srichuwong et al. 2005). The larger granule size of starch and higher peak viscosity of KS in the study confirmed this assumption. Furthermore, studies reported the relation between larger granule size of starch and greater peak viscosity (Zhang et al. 2018). Similar to this assumption, Dhital et al. (2011) showed that the peak viscosity of both maize and potato starch raised substantially with the increase in the granule size. Similarly, Kaur et al. (2007) reported that the granule size of starches obtained from three potato cultivars influences peak viscosity, with larger granule fractions having the highest peak viscosity and small granule fractions having the lowest.

**FIGURE 2:**
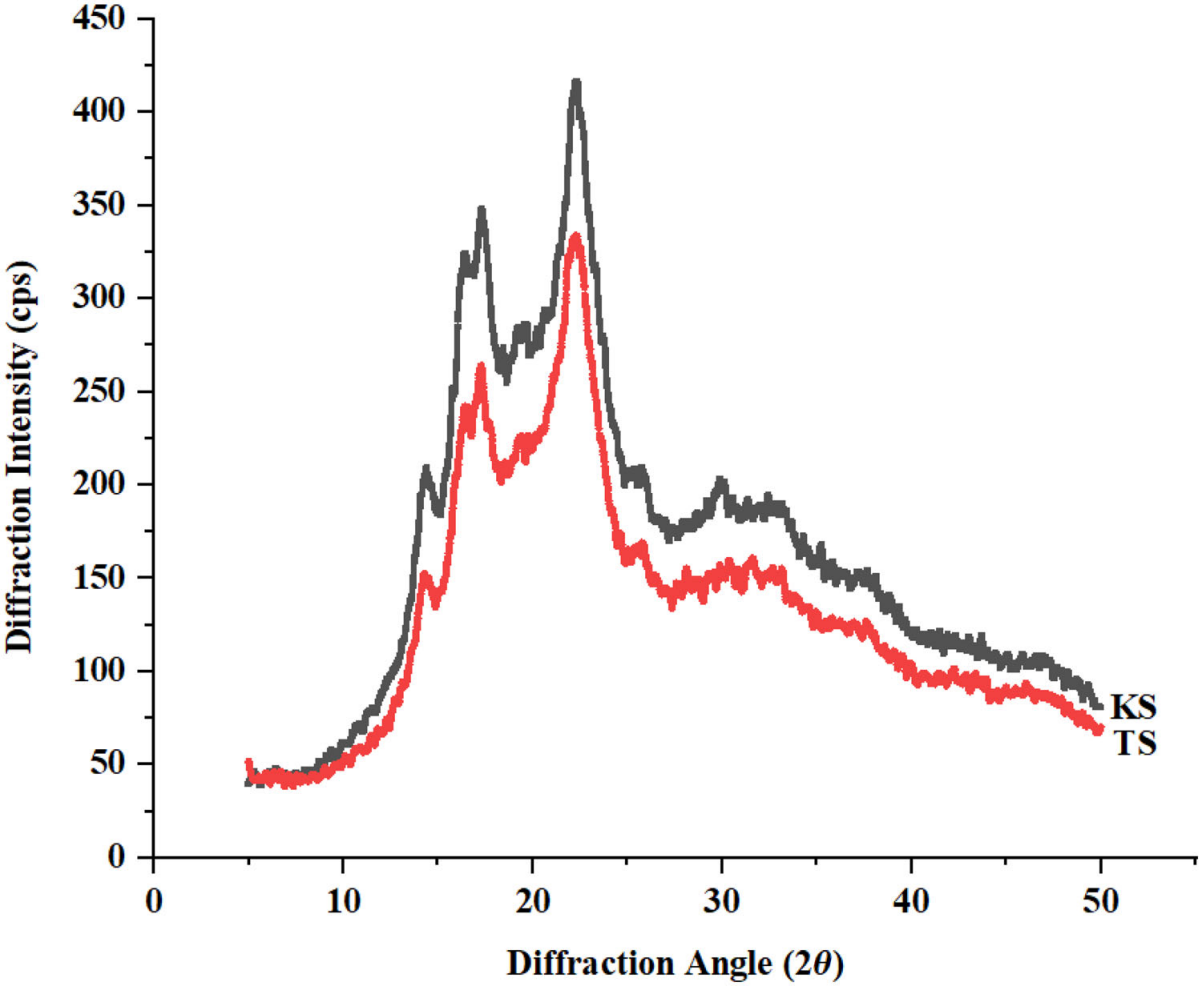
X‐ray diffraction patterns of buckwheat starch (KS and TS).

**TABLE 4:** Pasting properties of buckwheat starch (KS and TS).

Pasting properties	Buckwheat starch	
	KS	TS
Pasting temperature (°C)	71.00 ± 0.42^a^	71.44 ± 0.05^a^
Peak viscosity (BU)	743.55 ± 0.91^a^	702.20 ± 0.28^b^
End‐of‐cooling period viscosity (BU)	974.25 ± 0.35^a^	942.50 ± 1.55^b^
Breakdown (BU)	76.00 ± 1.41^a^	67.70 ± 2.26^b^
Setback (BU)	333.55 ± 4.87^a^	305.00 ± 4.66^b^

Data presented as mean ± standard deviation. Mean values within the same row with different uppercase superscript letters are significantly different (*p* < 0.05).

Also, the final viscosity of KS (974.25 ± 0.35 BU) was significantly higher than TS (942.50 ± 1.55 BU), which could be described by the fact that KS has greater amylose content and amylose leaches out into the water phase between the granules, making it more difficult for the granules to swell (Mauro et al. 2023).

The temperature at which starch viscosity begins to increase is known as the starch pasting temperature. Table 4 indicates nonsignificant difference in the pasting temperatures between KS and TS. A statistically notable difference was noted in the breakdown and setback viscosities of KS and TS. The lower setback viscosity of WS is helpful for baked foods, which indicates that it might have lesser retrogradation which retards staling (Aluwi et al. 2017). Also, the lower breakdown viscosity of TS indicates its resistance to breakdown as well as the alteration in viscosity during heating induced by shear (Nalbandian et al. 2022)

### 3.9 Correlation Between Structural and Functional Properties of Buckwheat Starch

**FIGURE 5:**
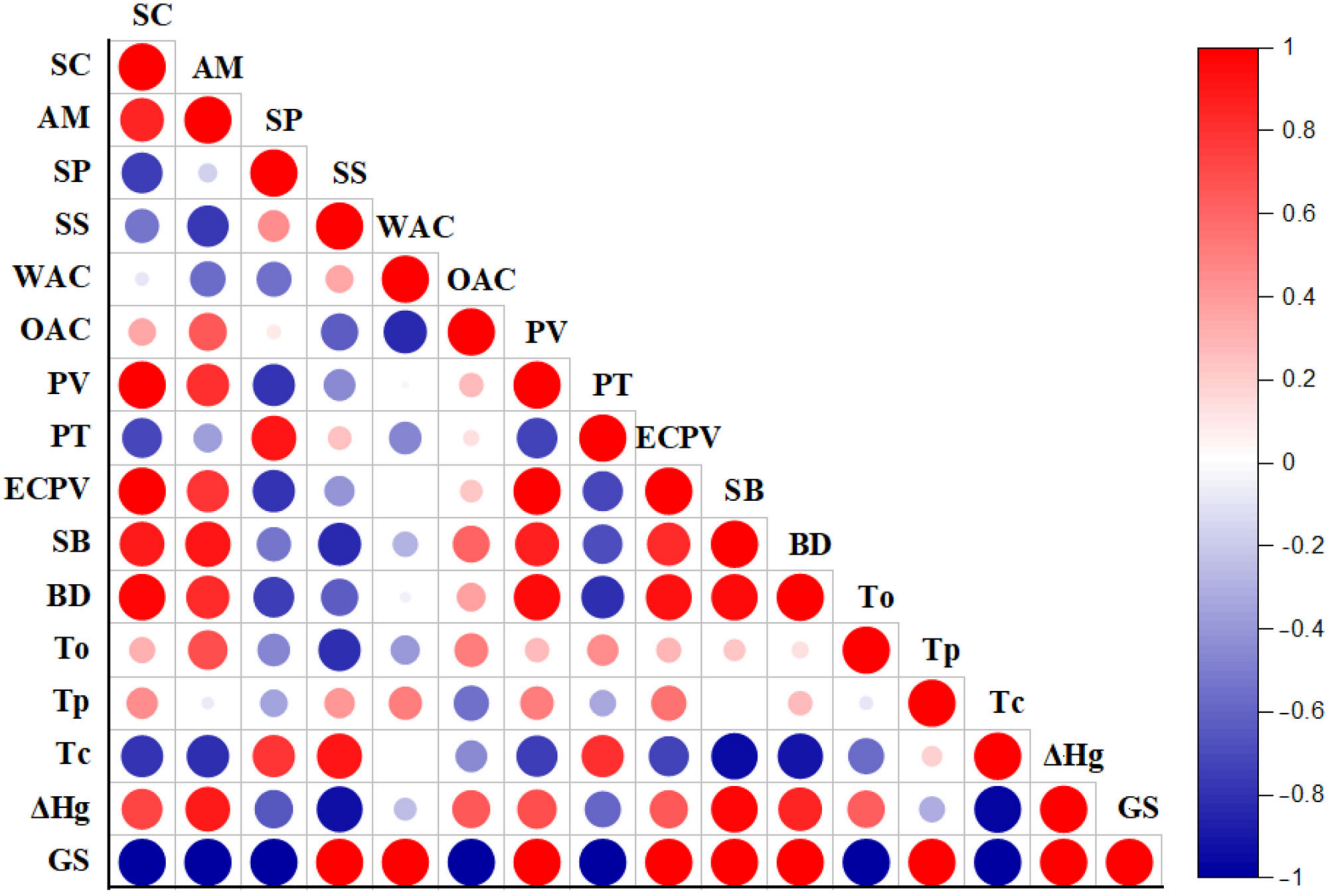
Pearson correlation analysis between structural, functional, and physicochemical profile of buckwheat starch (KS and TS). AM, amylose content; BD, breakdown; ECPV, end‐of‐cooling period viscosity; OAC, oil absorption capacity; PT, pasting temperature; PV, peak viscosity; SB, setback; SC, starch content; SP, swelling power; SS, solubility; *T*_o_, *T*_p_, *T*_c_, transition temperatures (*T*_o_, onset temperature; *T*_p_, peak temperature; *T*_c_, concluding temperature); WAC, water absorption capacity; Δ*H*_g_, gelatinization enthalpy change; GS, granule size.

Pearson correlation analysis was carried out to examine the correlation among the structural and functional attributes of buckwheat starch (KS and TS; Figure 5). Pearson correlation exhibited a strong correlation among starch content and peak viscosity (*r* = 0.99), end‐of‐cooling period viscosity (*r* = 0.99), breakdown (*r* = 0.96), setback (*r* = 0.89), and amylose content (*r* = 0.84). Conversely, starch content showed a negative correlation with swelling power (*r* = −0.74), water solubility (*r* = −0.52), and water absorption capacity (*r* = −0.08). This indicates that when the concentration of starch in flour is high, its capacity to expand, dissolve, and soak up water diminishes; therefore, the higher the starch amount, the lower its ability to interact with water (Mauro et al. 2023). Moreover, the observed negative correlation between starch content and swelling power may be attributed to the high amylose content in buckwheat starches and the linear arrangement of amylose molecules within the starch granules (Gao et al. 2023). Furthermore, Amylose content displayed a notable positive association with both setback viscosity (*r* = 0.91) and gelatinization enthalpy change (*r* = 0.88). Setback viscosity results from amylose molecules forming crystalline structures as a starch paste cools down, leading to an increase in viscosity. Thus, increased amylose levels result in a higher setback viscosity (Tao et al. 2019). Moreover, the correlation analysis indicated that there is a positive association among the starch solubility and the final gelatinization temperature. This suggests that when starch is heated past a certain gelatinization temperature, it absorbs water and swells, resulting in higher solubility and a gel‐like texture. Essentially, as the starch undergoes gelatinization, its solubility greatly increases (Kadam et al. 2015).

Furthermore, a significant inverse correlation was noted among starch peak viscosity and final gelatinization temperature (*r* = 0.90), indicating that the higher the temperature required for complete starch gelatinization, the lower the peak viscosity it will achieve when heated (Singh et al. 2006). Additionally, the thickness of a starch pastes after it has cooled was found to have a direct relationship with the setback measurement. As starch molecules cool, they realign and create a more organized structure, which results in higher viscosity. A greater setback results in increased retrogradation tendency and greater syneresis (Yılmaz Tuncel et al. 2022).

## 4 Conclusion

Starch isolated from different buckwheat varieties showcased diverse physicochemical and functional attributes. KS exhibited higher starch purity and amylose content. In terms of functional properties, both KS and TS demonstrated an increasing trend in water solubility and swelling power as temperatures rose from 30 to 90°C. A higher gelatinization enthalpy of buckwheat starch, particularly (KS), signified a higher energy needed for melting starch granules. Furthermore, enthalpies of retrogradation (Δ*H*_r_) for KS and TS were significantly less than that of gelatinized starch which was correlated with amylose content. A notable significant difference was observed in the peak viscosities of the two buckwheat starches. In context to the size of starch, KS and TS showed smaller sizes as compared to the common cereal starches. These characteristics significantly influence the use of starch as a thickener, binder, or texturizer in food products. The small size, high amylose content, and higher gelatinization transition temperatures (*T*_o_, *T*_p_, *T*_c_) of buckwheat starch make it suitable for usage in various food applications such as fat‐rich bakery products (due to small molecular size), ready‐to‐eat meals and frozen foods (due to high gelatinization temperature), and processed foods and encapsulant (due to high amylose content). However, further research will significantly enhance the usage of buckwheat starch in the food industry.

Additionally, analysis using Pearson correlation revealed a significant relationship between the starch content of buckwheat and its functional and structural characteristics. The findings of the study highlighted the significance of functional, morphological, pasting, and thermal characteristics of starch for its use in the food products. A detailed examination of the attributes of starch helps find its end uses.

## Author Contributions

**Shweta Suri**: writing–original draft, conceptualization, data curation, methodology, investigation, formal analysis, visualization. **Aniket Kamboj**: methodology, formal analysis, writing–review and editing. **Xiaofeng Guo**: writing–review and editing, methodology, formal analysis, data curation. **Kevin M. Murphy**: writing–review and editing, resources. **Girish M. Ganjyal**: funding acquisition, conceptualization, supervision, resources, project administration, writing–review and editing.

## Conflicts of Interest

The authors declare no conflicts of interest.

## References

[jfds70219-bib-0001] AACC . 2005. Approved Methods of the AACC. American Association of Cereal Chemists.

[jfds70219-bib-0002] Abd Karim, A. , M. H. Norziah , and C. C Seow . 2000. “Methods for the Study of Starch Retrogradation.” Food Chemistry 71, no. 1: 9–36. 10.1016/S0308-8146(00)00130-8.

[jfds70219-bib-0003] Ahmed, A. , N. Khalid , A. Ahmad , N. A. Abbasi , M. S. Z. Latif , and M. A. Randhawa . 2014. “Phytochemicals and Biofunctional Properties of Buckwheat: A Review.” The Journal of Agricultural Science 152, no. 3: 349–369. 10.1017/S0021859613000166.

[jfds70219-bib-0004] Aluwi, N. A. , K. M. Murphy , and G. M. Ganjyal . 2017. “Physicochemical Characterization of Different Varieties of Quinoa.” Cereal Chemistry 94, no. 5: 847–856. 10.1094/CCHEM-10-16-0251-R.

[jfds70219-bib-0005] AOAC . 2006. Official Methods of Analysis of the Association of Official Analytical Chemists. Association of Official Analytical Chemists.

[jfds70219-bib-0006] Bajaj, R. , N. Singh , A. Ghumman , A. Kaur , and H. N. Mishra . 2022. “Effect of High Pressure Treatment on Structural, Functional, and In‐Vitro Digestibility of Starches From Tubers, Cereals, and Beans.” Starch‐Stärke 74, no. 1–2: 2100096. 10.1002/star.202100096.

[jfds70219-bib-0007] Ballester‐Sánchez, J. , J. V. Gil , M. T. Fernández‐Espinar , and C. M. Haros . 2019. “Quinoa Wet‐Milling: Effect of Steeping Conditions on Starch Recovery and Quality.” Food Hydrocolloids 89: 837–843. 10.1016/j.foodhyd.2018.11.053.

[jfds70219-bib-0008] Bernin, J. , P. Watanabe , C. E. Wagner , S. Smith , and G. M. Ganjyal . 2024. “Mung Bean Protein Enhances the Expansion of Corn Starch During Twin‐Screw Extrusion.” Journal of Food Science 89, no. 12: 9379–9391. 10.1111/1750-3841.17375. 39495597 PMC11673559

[jfds70219-bib-0010] Breslauer, R. , E. Nalbandian , T. Reinman , M. Rezaey , G. M. Ganjyal , and K. M. Murphy . 2023b. “Buckwheat Production and Value‐Added Processing: A Review of Potential Western Washington Cropping and Food System Applications.” Sustainability 15, no. 20: 14758. 10.3390/su152014758.

[jfds70219-bib-0011] Breslauer, R. , J. K. O'Dea , S. Bramwell , and K. M. Murphy . 2023a. Buckwheat Production West of the Cascades. PNW (Series), 732. Washington State University Extension. 10.7273/000006215.

[jfds70219-bib-0012] Christa, K. , M. Soral‐Śmietana , and G. Lewandowicz . 2009. “Buckwheat Starch: Structure, Functionality and Enzyme In Vitro Susceptibility Upon the Roasting Process.” International Journal of Food Sciences and Nutrition 60, no. sup4: 140–154. 10.1080/09637480802641288. 19194812

[jfds70219-bib-0013] Dhital, S. , A. K. Shrestha , J. Hasjim , and M. J. Gidley . 2011. “Physicochemical and Structural Properties of Maize and Potato Starches as a Function of Granule Size.” Journal of Agricultural and Food Chemistry 59, no. 18: 10151–10161. 10.1021/jf202293s. 21838326

[jfds70219-bib-0014] Dong, S. , G. Fang , Z. Luo , and Q. Gao . 2021. “Effect of Granule Size on the Structure and Digestibility of Jackfruit Seed Starch.” Food Hydrocolloids 120: 106964. 10.1016/j.foodhyd.2021.106964.

[jfds70219-bib-1014] Dura, A. , W. Błaszczak , and C. M. Rosell . 2014. “Functionality of Porous Starch Obtained by Amylase or Amyloglucosidase Treatments.” Carbohydrate Polymers 101: 837–845. 24299846 10.1016/j.carbpol.2013.10.013

[jfds70219-bib-0015] FAOSTAT . 2024. Food and Agriculture Data. Food and Agriculture Organization. FAO. Accessed October 7, 2024. https://www.fao.org/faostat/en/#data/QCL.

[jfds70219-bib-0016] Gao, J. , I. Kreft , G. Chao , et al. 2016. “Tartary Buckwheat (*Fagopyrum tataricum* Gaertn.) Starch, a Side Product in Functional Food Production, as a Potential Source of Retrograded Starch.” Food Chemistry 190: 552–558. 10.1016/j.foodchem.2015.05.122. 26213009

[jfds70219-bib-0017] Gao, L. , G. Haesaert , F. Van Bockstaele , P. Vermeir , A. Skirtach , and M. Eeckhout . 2024. “Combined Effects of Nitrogen and Sulfur Fertilizers on Chemical Composition, Structure and Physicochemical Properties of Buckwheat Starch.” Food Chemistry 459: 140351. 10.1016/j.foodchem.2024.140351. 38981377

[jfds70219-bib-0018] Gao, L. , F. Van Bockstaele , B. Lewille , G. Haesaert , and M. Eeckhout . 2023. “Characterization and Comparative Study on Structural and Physicochemical Properties of Buckwheat Starch From 12 Varieties.” Food Hydrocolloids 137: 108320. 10.1016/j.foodhyd.2022.108320.

[jfds70219-bib-0019] Gao, L. , M. Xia , Z. Li , et al. 2020. “Common Buckwheat‐Resistant Starch as a Suitable Raw Material for Food Production: A Structural and Physicochemical Investigation.” International Journal of Biological Macromolecules 145: 145–153. 10.1016/j.ijbiomac.2019.12.116. 31846660

[jfds70219-bib-0020] Giménez‐Bastida, J. A. , M. Piskuła , and H. Zieliński . 2015. “Recent Advances in Development of Gluten‐Free Buckwheat Products.” Trends in Food Science & Technology 44, no. 1: 58–65. 10.1016/j.tifs.2015.02.013.

[jfds70219-bib-0021] Goel, C. , A. D. Semwal , A. Khan , S. Kumar , and G. K. Sharma . 2020. “Physical Modification of Starch: Changes in Glycemic Index, Starch Fractions, Physicochemical and Functional Properties of Heat‐Moisture Treated Buckwheat Starch.” Journal of Food Science and Technology 57: 2941–2948. 10.1007/s13197-020-04326-4. 32624599 PMC7316914

[jfds70219-bib-0022] Gregori, M. , and I. Kreft . 2012. “Breakable Starch Granules in a Low‐Amylose Buckwheat (*Fagopyrum esculentum* Moench) Mutant.” Journal of Food, Agriculture & Environment 10, no. 2: 258–262.

[jfds70219-bib-0023] Hatcher, D. W. , S. You , J. E. Dexter , C. Campbell , and M. S. Izydorczyk . 2008. “Evaluation of the Performance of Flours From Cross‐and Self‐Pollinating Canadian Common Buckwheat (*Fagopyrum esculentum* Moench) Cultivars in Soba Noodles.” Food Chemistry 107, no. 2: 722–731. 10.1016/j.foodchem.2007.08.072.

[jfds70219-bib-0024] Hu, J. , X. Li , Z. Cheng , et al. 2022. “Modified Tartary Buckwheat (*Fagopyrum tataricum* Gaertn.) Starch by Gaseous Ozone: Structural, Physicochemical and In Vitro Digestible Properties.” Food Hydrocolloids 125: 107365. 10.1016/j.foodhyd.2021.107365.

[jfds70219-bib-0025] Huang, J. , Z. Wang , L. Fan , and S. Ma . 2022. “A Review of Wheat Starch Analyses: Methods, Techniques, Structure and Function.” International Journal of Biological Macromolecules 203: 130–142. 10.1016/j.ijbiomac.2022.01.149. 35093434

[jfds70219-bib-0026] Huo, D. , X. Xiao , X. Zhang , X. Hao , Z. Hao , and E. Li . 2023. “Exploration of Unique Starch Physicochemical Properties of Novel Buckwheat Lines Created by Crossing Golden Buckwheat and Tatary Buckwheat.” Food Chemistry: X 20: 100949. 10.1016/j.fochx.2023.100949. 38144746 PMC10739759

[jfds70219-bib-0027] Jha, R. , K. Zhang , Y. He , et al. 2024. “Global Nutritional Challenges and Opportunities: Buckwheat, a Potential Bridge Between Nutrient Deficiency and Food Security.” Trends in Food Science & Technology 145: 104365. 10.1016/j.tifs.2024.104365.

[jfds70219-bib-0028] Kadam, S. U. , B. K. Tiwari , and C. P. O'Donnell . 2015. “Improved Thermal Processing for Food Texture Modification.” In Modifying Food Texture *Volume 1: Novel Ingredients and Processing Techniques* , edited by J. Chen and A. Rosenthal , 115–131. Woodhead Publishing Series in Food Science, Technology and Nutrition. 10.1016/B978-1-78242-333-1.00006-1.

[jfds70219-bib-0029] Kaur, L. , J. Singh , O. J. McCarthy , and H. Singh . 2007. “Physico‐Chemical, Rheological and Structural Properties of Fractionated Potato Starches.” Journal of Food Engineering 82, no. 3: 383–394. 10.1016/j.jfoodeng.2007.02.059.

[jfds70219-bib-0030] Kim, B. S. , H. S. Kim , J. S. Hong , K. C. Huber , J. H. Shim , and S. H. Yoo . 2013. “Effects of Amylosucrase Treatment on Molecular Structure and Digestion Resistance of Pre‐Gelatinised Rice and Barley Starches.” Food Chemistry 138, no. 2–3: 966–975. 10.1016/j.foodchem.2012.11.028. 23411202

[jfds70219-bib-0031] Li, C. , R. J. Kowalski , L. Li , and G. M. Ganjyal . 2017. “Extrusion Expansion Characteristics of Samples of Select Varieties of Whole Yellow and Green Dry Pea Flours.” Cereal Chemistry 94, no. 3: 385–391. 10.1094/CCHEM-04-16-0079-R.

[jfds70219-bib-0032] Li, S. Q. , and Q. H. Zhang . 2001. “Advances in the Development of Functional Foods From Buckwheat.” Critical Reviews in Food Science and Nutrition 41, no. 6: 451–464. 10.1080/20014091091887. 11592684

[jfds70219-bib-0033] Li, W. , F. Cao , J. Fan , et al. 2014. “Physically Modified Common Buckwheat Starch and Their Physicochemical and Structural Properties.” Food Hydrocolloids 40: 237–244. 10.1016/j.foodhyd.2014.03.012.

[jfds70219-bib-0034] Lin, J. , S. Fan , Y. Ruan , et al. 2023. “Tartary Buckwheat Starch Modified With Octenyl Succinic Anhydride for Stabilization of Pickering Nanoemulsions.” Foods 12, no. 6: 1126. 10.3390/foods12061126. 36981053 PMC10048578

[jfds70219-bib-0035] Liu, H. , L. Wang , R. Cao , H. Fan , and M. Wang . 2016. “In Vitro Digestibility and Changes in Physicochemical and Structural Properties of Common Buckwheat Starch Affected by High Hydrostatic Pressure.” Carbohydrate Polymers 144: 1–8. 10.1016/j.carbpol.2016.02.028. 27083786

[jfds70219-bib-0036] Lu, L. , and B. K. Baik . 2015. “Starch Characteristics Influencing Resistant Starch Content of Cooked Buckwheat Groats.” Cereal Chemistry 92, no. 1: 65–72. 10.1094/CCHEM-04-14-0062-R.

[jfds70219-bib-0037] Luo, K. , X. Zhou , and G. Zhang . 2019. “The Impact of Tartary Buckwheat Extract on the Nutritional Property of Starch in a Whole Grain Context.” Journal of Cereal Science 89: 102798. 10.1016/j.jcs.2019.102798.

[jfds70219-bib-0038] Ma, Q. , Y. Yu , Z. Zhou , L. Wang , and R. Cao . 2023. “Effects of Different Treatments on Composition, Physicochemical and Biological Properties of Soluble Dietary Fiber in Buckwheat Bran.” Food Bioscience 53: 102517. 10.1016/j.fbio.2023.102517.

[jfds70219-bib-0039] Malumba, P. , C. Massaux , C. Deroanne , T. Masimango , and F. Béra . 2009. “Influence of Drying Temperature on Functional Properties of Wet‐Milled Starch Granules.” Carbohydrate Polymers 75, no. 2: 299–306. 10.1016/j.carbpol.2008.07.027.

[jfds70219-bib-0041] Mauro, R. R. , A. J. Vela , and F. Ronda . 2023. “Impact of Starch Concentration on the Pasting and Rheological Properties of Gluten‐Free Gels. Effects of Amylose Content and Thermal and Hydration Properties.” Foods 12, no. 12: 2281. 10.3390/foods12122281. 37372492 PMC10297029

[jfds70219-bib-0042] Megazyme . 2024. Amylose/Amylopectin Assay Kit. Accessed from https://www.megazyme.com/amylose‐amylopectin‐assay‐kit/. (Accessed October, 25 2024)

[jfds70219-bib-0043] Nalbandian, E. , E. Pietrysiak , K. M. Murphy , and G. M. Ganjyal . 2022. “Different Breeding Lines of Quinoa Significantly Influence the Quality of Baked Cookies and Cooked Grains.” Journal of Food Science 87, no. 12: 5225–5239. 36331266 10.1111/1750-3841.16354

[jfds70219-bib-0044] Neethirajan, S. , K. Tsukamoto , H. Kanahara , and S. Sugiyama . 2012. “Ultrastructural Analysis of Buckwheat Starch Components Using Atomic Force Microscopy.” Journal of Food Science 77, no. 1: N2–N7. 10.1111/j.1750-3841.2011.02442.x. 22260119

[jfds70219-bib-0045] Pietrysiak, E. , A. Zak , M. Ikuse , et al. 2024. “Impact of Genotypic Variation and Cultivation Conditions on the Techno‐Functional Characteristics and Chemical Composition of 25 New Canadian Quinoa Cultivars.” Food Research International 195: 114903. 10.1016/j.foodres.2024.114903. 39277215

[jfds70219-bib-0046] Pietrzak, W. , and J. Kawa‐Rygielska . 2021. “Effect of Sieving and Alkaline Extraction of Whole Rye Meal on the Production of Ethanol and Valuable By‐Products in an Integrated Bioprocess.” Journal of Cereal Science 102: 103342. 10.1016/j.jcs.2021.103342.

[jfds70219-bib-0047] Qin, P. , Q. Wang , F. Shan , Z. Hou , and G. Ren . 2010. “Nutritional Composition and Flavonoids Content of Flour From Different Buckwheat Cultivars.” International Journal of Food Science & Technology 45, no. 5: 951–958. 10.1111/j.1365-2621.2010.02231.x.

[jfds70219-bib-0048] Rainakari, A. I. , H. Rita , T. Putkonen , and H. Pastell . 2016. “New Dietary Fibre Content Results for Cereals in the Nordic Countries Using AOAC 2011.25 Method.” Journal of Food Composition and Analysis 51: 1–8. 10.1016/j.jfca.2016.06.001.

[jfds70219-bib-0049] Rodriguez‐Sandoval, E. , I. Prasca‐Sierra , and V. Hernandez . 2017. “Effect of Modified Cassava Starch as a Fat Replacer on the Texture and Quality Characteristics of Muffins.” Journal of Food Measurement and Characterization 11: 1630–1639. 10.1007/s11694-017-9543-0.

[jfds70219-bib-0051] Sindhu, R. , and B. S. Khatkar . 2023. “Influence of Oxidation, Acetylation and Hydrothermal Treatment on Structure and Functionality of Common Buckwheat Starch.” International Journal of Biological Macromolecules 253: 127211. 10.1016/j.ijbiomac.2023.127211. 37797848

[jfds70219-bib-0052] Singh, N. , L. Kaur , K. S. Sandhu , J. Kaur , and K. Nishinari . 2006. “Relationships Between Physicochemical, Morphological, Thermal, Rheological Properties of Rice Starches.” Food Hydrocolloids 20, no. 4: 532–542. 10.1016/j.foodhyd.2005.05.003.

[jfds70219-bib-0053] Singh, N. , J. Singh , L. Kaur , N. S. Sodhi , and B. S. Gill . 2003. “Morphological, Thermal and Rheological Properties of Starches From Different Botanical Sources.” Food Chemistry 81, no. 2: 219–231. 10.1016/S0308-8146(02)00416-8.

[jfds70219-bib-0054] Sofi, S. A. , N. Ahmed , A. Farooq , et al. 2022. “Nutritional and Bioactive Characteristics of Buckwheat, and Its Potential for Developing Gluten‐Free Products: An Updated Overview.” Food Science & Nutrition 11, no. 5: 2256–2276. 10.1002/fsn3.3166. 37181307 PMC10171551

[jfds70219-bib-1054] Srichuwong, S. , and J. I. Jane . 2007. “Physicochemical Properties of Starch Affected by Molecular Composition and Structures: A Review.” Food Science and Biotechnology 16, no. 5: 663–674.

[jfds70219-bib-0055] Srichuwong, S. , T. C. Sunarti , T. Mishima , N. Isono , and M. Hisamatsu . 2005. “Starches From Different Botanical Sources II: Contribution of Starch Structure to Swelling and Pasting Properties.” Carbohydrate Polymers 62, no. 1: 25–34. 10.1016/j.carbpol.2005.07.003.

[jfds70219-bib-0056] Suzuki, T. , T. Noda , T. Morishita , K. Ishiguro , S. Otsuka , and A. Brunori . 2020. “Present Status and Future Perspectives of Breeding for Buckwheat Quality.” Breeding Science 70, no. 1: 48–66. 10.1270/jsbbs.19018. 32351304 PMC7180147

[jfds70219-bib-0057] Tamura, M. , Y. Yoshimura , T. Saito , and T. Koyama . 2021. “Comparison of Standard and Non‐Standard Buckwheat Groats for Cooking, Physicochemical and Nutritional Properties, and In Vitro Starch Digestibility.” Future Foods 3: 100029. 10.1016/j.fufo.2021.100029.

[jfds70219-bib-0058] Tao, K. , C. Li , W. Yu , R. G. Gilbert , and E. Li . 2019. “How Amylose Molecular Fine Structure of Rice Starch Affects Functional Properties.” Carbohydrate Polymers 204: 24–31. 10.1016/j.carbpol.2018.09.078. 30366537

[jfds70219-bib-0059] Wronkowska, M. , and M. Haros . 2014. “Wet‐Milling of Buckwheat With Hull and Dehulled—The Properties of the Obtained Starch Fraction.” Journal of Cereal Science 60, no. 3: 477–483. 10.1016/j.jcs.2014.09.004.

[jfds70219-bib-0060] Yang, J. , Z. Gu , L. Zhu , et al. 2019. “Buckwheat Digestibility Affected by the Chemical and Structural Features of Its Main Components.” Food Hydrocolloids 96: 596–603. 10.1016/j.foodhyd.2019.06.001.

[jfds70219-bib-0061] Yang, P. , A. E. Haken , Y. Niu , et al. 2005. “Effect of Steeping With Sulfite Salts and Adjunct Acids on Corn Wet‐Milling Yields and Starch Properties.” Cereal Chemistry 82, no. 4: 420–424. 10.1094/CC-82-0420.

[jfds70219-bib-0062] Yılmaz Tuncel, N. , F. Korkmaz , H. Polat , and N. B. Tuncel . 2022. “Monitoring Starch Hydrolysis With Micro Visco‐Amylo‐Graph for the Production of Chickpea Milk and Optimization of the Parameters With Response Surface Methodology.” Journal of Food Science and Technology 59: 1–10. 10.1007/s13197-021-05332-w. 35875212 PMC9304489

[jfds70219-bib-0063] Yoshimoto, Y. , T. Egashira , I. Hanashiro , H. Ohinata , Y. Takase , and Y. Takeda . 2004. “Molecular Structure and some Physicochemical Properties of Buckwheat Starches.” Cereal Chemistry 81, no. 4: 515–520. 10.1094/CCHEM.2004.81.4.515.

[jfds70219-bib-0064] Yun, S. H. , and N. K. Matheson . 1990. “Estimation of Amylose Content of Starches After Precipitation of Amylopectin by Concanavalin‐A.” Starch‐Stärke 42, no. 8: 302–305. 10.1002/star.19900420805.

[jfds70219-bib-0066] Zhang, B. , X. Li , J. Liu , F. Xie , and L. Chen . 2013. “Supramolecular Structure of A‐and B‐type Granules of Wheat Starch.” Food Hydrocolloids 31, no. 1: 68–73. 10.1016/j.foodhyd.2012.10.006.

[jfds70219-bib-0067] Zhang, L. , Y. Zhao , W. Hu , et al. 2018. “Multi‐Scale Structures of Cassava and Potato Starch Fractions Varying in Granule Size.” Carbohydrate Polymers 200: 400–407. 10.1016/j.carbpol.2018.08.022. 30177180

[jfds70219-bib-0068] Zheng, F. , Q. Xu , S. Zeng , et al. 2023. “Multi‐Scale Structural Characteristics of Black Tartary Buckwheat Resistant Starch by Autoclaving Combined With Debranching Modification.” International Journal of Biological Macromolecules 249: 126102. 10.1016/j.ijbiomac.2023.126102. 37541464

[jfds70219-bib-0069] Zheng, G. H. , F. W. Sosulski , and R. T. Tyler . 1997. “Wet‐Milling, Composition and Functional Properties of Starch and Protein Isolated From Buckwheat Groats.” Food Research International 30, no. 7: 493–502. 10.1016/S0963-9969(98)00021-0.

[jfds70219-bib-0070] Zhou, M. , Y. U. Tang , X. Deng , et al. 2018. “Overview of Buckwheat Resources in the World.” In Buckwheat Germplasm in the World, edited by M. Zhou , G. Suvorova and S. H. Woo , 1–7. Academic Press. 10.1016/B978-0-12-811006-5.00001-X.

[jfds70219-bib-0071] Zhu, F. 2016a. “Buckwheat Starch: Structures, Properties, and Applications.” Trends in Food Science & Technology 49: 121–135. 10.1016/j.tifs.2015.12.002.

[jfds70219-bib-0072] Zhu, F. 2016b. “Chemical Composition and Health Effects of Tartary Buckwheat.” Food Chemistry 203: 231–245. 10.1016/j.foodchem.2016.02.050. 26948610

